# Phylogenetic and functional analysis of the Cation Diffusion Facilitator (CDF) family: improved signature and prediction of substrate specificity

**DOI:** 10.1186/1471-2164-8-107

**Published:** 2007-04-23

**Authors:** Barbara Montanini, Damien Blaudez, Sylvain Jeandroz, Dale Sanders, Michel Chalot

**Affiliations:** 1 UMR INRA/UHP 1136 Interactions Arbres/Micro-organismes, Nancy-Université, BP 239, 54506 Vandoeuvre, France; 2 Dipartimento di Biochimica e Biologia Molecolare, Università di Parma, Viale G. P. Usberti 23/A, 43100 Parma, Italy; 3 Biology Department, Area 9, University of York, PO Box 373, York YO10 5YW, UK

## Abstract

**Background:**

The Cation Diffusion Facilitator (CDF) family is a ubiquitous family of heavy metal transporters. Much interest in this family has focused on implications for human health and bioremediation. In this work a broad phylogenetic study has been undertaken which, considered in the context of the functional characteristics of some fully characterised CDF transporters, has aimed at identifying molecular determinants of substrate selectivity and at suggesting metal specificity for newly identified CDF transporters.

**Results:**

Representative CDF members from all three kingdoms of life (Archaea, Eubacteria, Eukaryotes) were retrieved from genomic databases. Protein sequence alignment has allowed detection of a modified signature that can be used to identify new hypothetical CDF members. Phylogenetic reconstruction has classified the majority of CDF family members into three groups, each containing characterised members that share the same specificity towards the principally-transported metal, i.e. Zn, Fe/Zn or Mn. The metal selectivity of newly identified CDF transporters can be inferred by their position in one of these groups. The function of some conserved amino acids was assessed by site-directed mutagenesis in the poplar Zn^2+ ^transporter PtdMTP1 and compared with similar experiments performed in prokaryotic members. An essential structural role can be assigned to a widely conserved glycine residue, while aspartate and histidine residues, highly conserved in putative transmembrane domains, might be involved in metal transport. The potential role of group-conserved amino acid residues in metal specificity is discussed.

**Conclusion:**

In the present study phylogenetic and functional analyses have allowed the identification of three major substrate-specific CDF groups. The metal selectivity of newly identified CDF transporters can be inferred by their position in one of these groups. The modified signature sequence proposed in this work can be used to identify new hypothetical CDF members.

## Background

Copper, iron, zinc, cobalt, nickel and manganese are essential metal cations in cellular processes, since they act as important cofactors for many enzymes, are components of transcription factors and other proteins, and are essential for both mitochondrial and chloroplast functions. However, when present at high concentration, along with non-essential metals such as cadmium, mercury, silver and lead, essential metals can become extremely toxic, since they can cause oxidative damages or compete with other essential ions. A network of uptake, extrusion, chelation, trafficking and storage mechanisms ensures the maintenance of metal homeostasis at the cellular level. Specific transporters, encoded by multigenic families, are responsible for the uptake and secretion of metal ions, and for their sequestration into organelles [1-4].

Cation diffusion facilitator (CDF, TC 2.A.4) transporters, first identified by Nies and Silver [5], are ubiquitous, spanning all three kingdoms of life: Archaea, Eubacteria and Eukaryotes. Studies on CDF transporters may inform strategies for bioremediation and human nutrition and health [1,2,4]. All known CDF substrates are divalent metal cations with ionic radii of 72 (Zn^2+^) to 97 (Cd^2+^) pm [4]. Most CDFs are Me^2+^/H^+ ^(K^+^) antiporters [6-9] that catalyse the efflux of transition metal cations, including Zn^2+^, Co^2+^, Fe^2+^, Cd^2+^, Ni^2+^, or Mn^2+^, from the cytoplasm to the outside of the cell or into subcellular compartments [1,2,4,7,10-13]. The majority of CDF proteins possess six putative transmembrane domains (TMDs), with cytoplasmic N and C termini, as experimentally demonstrated for bacterial members [10,14]. A signature sequence between TMDs I and II was proposed in 1997 [15], and a characteristic C-terminal cation efflux domain is present in all members [16] (Pfam 01545, [17]). The most conserved regions are the amphipathic TMDs I, II, V and VI, which are likely involved in metal transfer [18]. However, some CDF members exhibit different types of secondary structure: for example the Msc2 protein of *Saccharomyces cerevisiae *is predicted to have 12 TMDs [19], and the human protein ZnT5 and its splice variant ZTL1 display 15 and 12 TMD topology, respectively [20,21]. Plant CDF members are usually called Metal Tolerance Proteins (MTPs), while vertebrate members are named Zinc Transporter (ZnT) or Solute carrier family 30 (SLC30).

Most CDF family transporters also contain a histidine-rich region, either between TMDs IV and V, or at the N and/or C termini. Such regions are predicted to be cytoplasmic, cis to metal uptake, and could function as potential metal (Zn^2+^, Co^2+^, and/or Cd^2+^) binding domains. However the ER-localised Zrg17, a Zn^2+ ^transporter recently characterised in yeast, displays a histidine-rich loop predicted to reside between TMDs III and IV toward the ER lumen [22]. The plant CDF member ShMTP1 (ShCDF8) isolated from *Stylosanthes hamata*, is involved in Mn^2+ ^homeostasis and tolerance, shows a predicted 4–5 TMD topology, and does not contain the histidine-rich domain [11,16].

Some CDF transporters function as homo-oligomeric complexes [23-26] or hetero-oligomeric complexes [22,24], and may directly interact with other proteins to regulate their activity by means of metal release to these proteins [27]. Indirect evidence that CDF transporters interact with other proteins also comes from studies on the rat ZnT4 [28].

Research over the past few years, mainly performed on prokaryotic CDF members, has aimed at identifying molecular mechanisms involved in metal binding and transport across the membranes. Key functional amino acid residues, identified by site-directed mutagenesis, reside in the three amphipathic and conserved transmembrane helices II, V and VI, which are supposed to constitute an inner core forming a channel [14,18,29,30]. Indeed recent studies on the CDF transporter EcFieF (YiiP) from *Escherichia coli *localised a substrate binding site at the dimer interface created by the TMDs II and V [31].

Here we undertake a phylogenetic analysis of CDF family amino acid sequences based on a set of CDF sequences retrieved from databases. Based on a multiple sequence alignment, we propose a modified signature for the CDF family that takes account of newly characterised members. Coupled with the substrate specificities of some characterised transporters, we are able to classify the family members into three major groups that have different selectivity towards the principally-transported metal (Zn-, Fe/Zn- and Mn-CDF). Functional analyses, based on site-directed mutagenesis, were carried out on the eukaryotic Zn^2+ ^transporter PtdMTP1 [23] to identify key residues important for CDF function. The role in metal selectivity of the group-conserved residue D (for Mn-specific CDF transporters) or H (for Zn- and Fe/Zn-CDF transporters) embedded in TMD V is also discussed.

## Results

### General features of the CDF family

#### Data mining and identification of an improved signature for the CDF family

Deduced amino acid sequences, belonging to characterised or hypothetical CDF transporters, have been retrieved from Genebank and SwissProt and from a set of annotated genomes, thus covering most phylogenetic groups (see Methods). An additional set of sequences, homologous to Zrg17 from *S. cerevisiae *was retrieved by a BLASTP search on completely sequenced genomes, since in the automatically annotated genomes these members were not identified.

Sequences that displayed significant truncation were discarded. A final set of 273 representative CDF amino acid sequences (see accession numbers in additional file 1) was used for phylogenetic analysis using MEGA software (version 3.1) [32]. CDF members displayed the highest conservation in trans-membrane regions, especially in TMD II, where the following signature was previously defined for this family [15].

S-X-[ASG]-[LIVMT]_2_-[SAT]-[DA]-[SGAL]-[LIVFYA]-[HDN]-X_3_-D-X_2_-[AS]

X = any amino acid

[ ] = one of the amino acid between brackets is possible

Since 1997, new CDF transporters have been characterised or identified by similarity with known CDFs, and the number of recognised CDF family members has dramatically expanded, in large part due to data coming from automated annotated genomic sequences; the first signature was derived from only 13 CDF members and now appears too restrictive to allow the recognition of the newly identified CDF members. From the original signature, an improved signature was designed based on the amino acid multiple sequence alignment. Compared to the original signature, this pattern is extended on the C terminus side and includes a fully conserved glycine (underlined below) and the downstream five amino acids. The lack of the conserved glycine and the downstream amino acids in the signature pattern allows a false match with non-CDF proteins otherwise absent when using the complete modified signature. The pattern was subsequently checked against Swiss-Prot (release 50.2), TrEMBL (release 33.2) and PDB databases at ScanProsite [33].

[SC]-X-[ASG]-[LIVM]-[LIVMTAF]-[SATG]-[DAELSTY]-[SGALFMTV]-{DKNPQR}-[HDNEL]-X_3_-[DH]-X_2_-[ASGLNTI]-X_20–25_-G-X_2_-[KNQRSY]-X-[DEGLNPQRST]

X = any amino acid

[ ] = one of the amino acid between brackets is possible

{ } = any amino acid except those between braces

When scanned against the three available databases, the original signature [15] matched with a total of 366 sequences, while the new proposed signature matched with 930 hypothetical CDFs.

**Figure 1:**
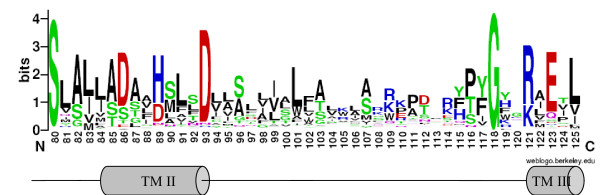
**CDF signature**. Sequence logo representation of the modified signature. The height of amino acid symbols is related to their conservation in the multiple alignment. Amino acid position refers to PtdMTP1. The hypothetical secondary structure (TMDs II and III) is shown below.

The tentative signature thus covers the second transmembrane helix, the start of the third TMD and the cytosolic loop connecting the two TMDs (Figure 1). The loop between TMD II and TMD III is represented by a non-conserved region of 20 to 25 residues; the CDF sequences retrieved by ScanProsite mostly carried 21 or 22 residues in that position (83% and 12% respectively). The signature matches with all those CDF sequences used in the alignment available at ScanProsite, with the exception of ScZrg17 from *S. cerevisiae *and some of its homologous sequences – recovered by BLASTP search on completely sequenced genomes-, which display a partial signature, since the first three amino acids are not conserved in these members. About 5% of matched sequences (48 out of 978) were false positive, since they clearly belonged to other families or because BLAST searches revealed that they did not match any of the sequence used for the alignment. These sequences mostly contained charged or large amino acid residues in the five free (X) positions around the conserved [DH] residue (position 89 in PtdMTP1, see Figure 1) that do not fit in TMD II of CDF members.

#### Identification of key residues important for the CDF function

**Figure 2:**
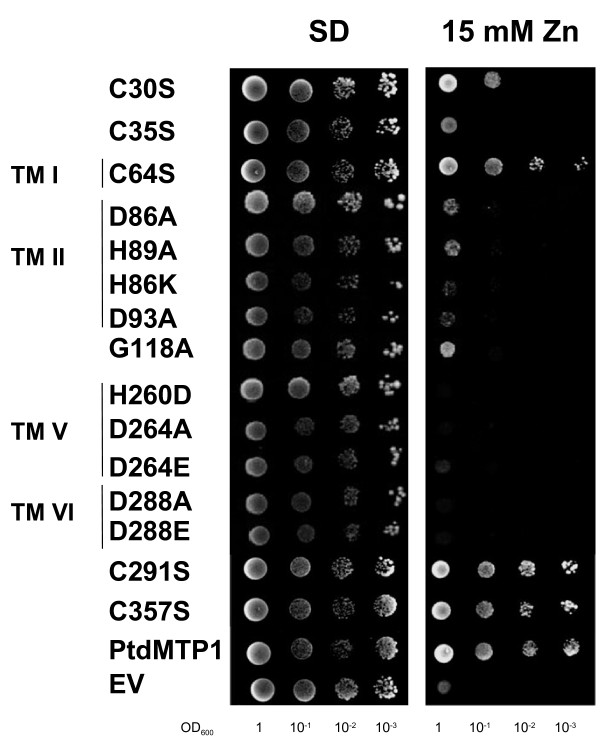
**Effect of amino acid substitution on the ability of PtdMTP1 to protect against Zn^2+ ^toxicity in yeast**. The Zn-hypersensitive yeast strain *zrc1*Δ, transformed with pYES2 bearing the mutated variants of PtdMTP1 was grown at 30°C in SD with 2% galactose (SD) until O.D. _600 nm _= 1. Serial dilutions were spotted on SD or on SD + 15 mM ZnCl_2 _(15 mM Zn). Photographs were taken after 6 days of growth at 30°C. For each mutation different yeast transformants were used and gave the same results. The amino acid substitutions along with their topological positions are shown on the left. EV: empty vector; PtdMTP1: wild type protein.

To delineate the importance of highly conserved residues, single amino acid substitutions were generated by site directed mutagenesis, on a well-characterised eukaryotic CDF, namely the vacuolar Zn^2+ ^transporter PtdMTP1 from the hybrid poplar *Populus trichocarpa *× *Populus deltoides *([23] and Table 1). The plant zinc transporter PtdMTP1 was chosen, since, to our knowledge, it is the only eukaryotic CDF for which various site directed mutagenesis data are already available [23]. The effect of individual amino acid changes was assessed by expressing mutated *PtdMTP1 *in the Zn-hypersensitive yeast mutant *zrc1*Δ and by monitoring Zn^2+ ^resistance of cells (Figure 2).

As suggested by topological predictions from diverse CDF family members, the highly conserved glycine residue resides in the cytoplasm close to the beginning of TMDIII. The G118A substitution led to a drastic decrease of function, as shown by a complementation test in the Zn-hypersensitive mutant *zrc1*Δ (Figure 2). Indeed, at 15 mM ZnCl_2_, only poor growth was measured even with a high plating density.

To check the localisation of the mutant protein, a GFP fused version was expressed in *zrc1*Δ. In common with the wild type PtdMTP1, the mutant G118A protein still localised on the tonoplast, and the loss of function was thus not due to a mis-localisation of the mutant protein. A number of mutagenesis analyses on prokaryotic zinc, or iron-zinc CDFs [14,29-31] did not include the substitution of this highly conserved glycine residue (Table 1).

From the sequence alignment, other amino acids were widely conserved, in particular charged and other polar residues in the amphipathic TMDs II, V and VI. These residues correspond, in PtdMTP1, to the aspartate residues located on TMDs II, V and VI (D86, D93, D264 and D288) and the histidine residues located on TMDs II and V (H89 and H260). The residues are likely to be involved in cation transport and/or binding; indeed when changed they affected the function of bacterial CDF members ([14,29-31] and Table 1). These six amino acids were individually substituted by alanine or by a residue that conserves the charge (glutamate instead of aspartate or lysine instead of histidine). Substitutions of all six residues resulted in the loss of PtdMTP1 function, in accord with the results of mutagenesis studies performed in prokaryotic members (Table 1 and [14,29-31]). None of the mutations tested displayed a hypersensitive phenotype, that is inhibition of growth at non toxic Zn^2+ ^concentration (≤ 10 mM ZnCl_2_). For D288A and D288E substitutions, vesicle-like localisations were observed, while the other PtdMTP1 variants still localised to the tonoplast.

### Phylogenetic tree of the CDF family

**Figure 3:**
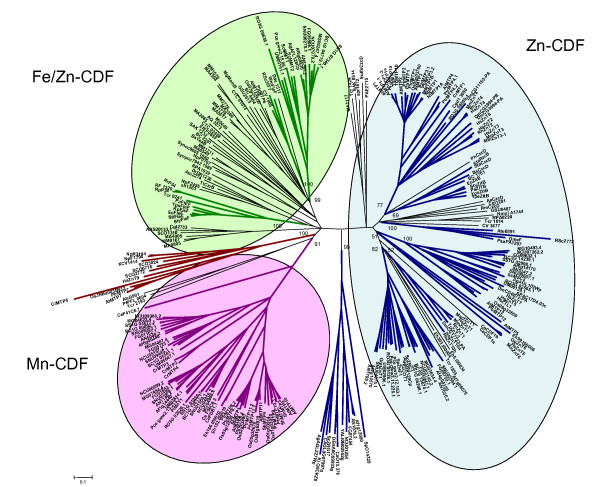
**Phylogenetic tree of the CDF family**. The Neighbor-Joining phylogenetic tree based on the protein alignment was created with MEGA 3.1. Clusters are represented with different colours with respect to metal specificity: blue is for zinc, green for iron/zinc, violet for manganese and brown for unknown specificity. The three CDF groups are surrounded with coloured boxes with the same colour-code. Bootstrap values are indicated for each cluster. The scale bar indicates an evolutionary distance of 0.1 amino acid substitution per site.

**Figure 4:**
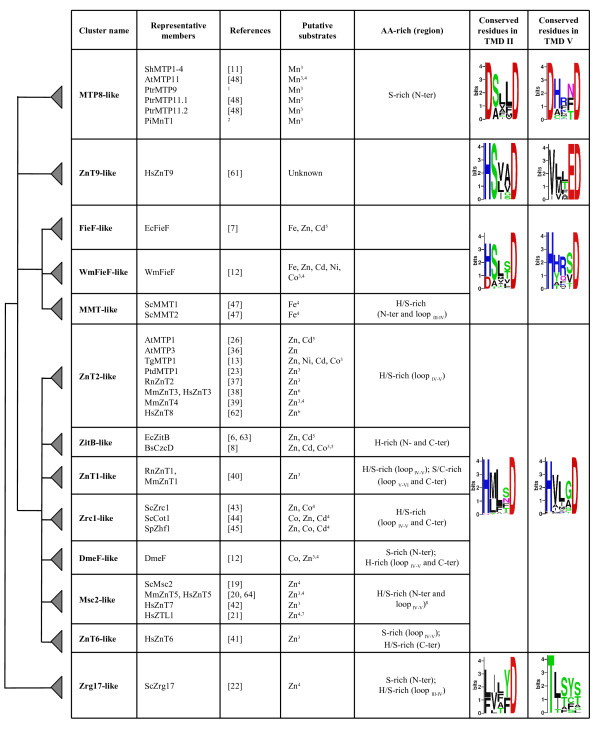
**Definition of CDF clusters and substrate specificities**. The characterised members along with their major substrate specificities, the corresponding references and histidine-rich (or serine/cysteine-rich) regions are indicated for each cluster. When more than one metal is transported, the metal preference is reflected by the written order within these metals. For each group and for Zrg17-like and ZnT9-like clusters logos showing the conserved residues in TMDs II and V are shown on the right. A simplified phylogenetic tree representation, expressed as a dendogram using Zrg17-like cluster as outgroup, is shown on the left. ^1 ^B. Montanini, D. Blaudez, M. Chalot, unpublished data; ^2 ^D. Blaudez, M. Chalot, unpublished data; ^3 ^assessed by heterologous complementation; ^4 ^deduced by mutant phenotype or over-expression in homologous system; ^5 ^measured in reconstituted proteoliposomes or in everted membrane vesicles; ^6 ^indirect evidence; ^7 ^measured in *Xenopus laevis *oocytes; ^8 ^by referring to the CDF domain.

Phylogenetic analysis using Neighbor-Joining (NJ) method was carried out on the 273-protein alignment, resulting in the tree shown in Figure 3 (see Methods). To avoid biased group distribution due to the extended N or C termini typical of some CDF transporters (e.g. ScMsc2, HsZTL1, HsZnT5), only the CDF domain was taken for the phylogenetic tree reconstruction. In particular, the start was set about 40 amino acids upstream from the first TMD, which corresponds to the first amino acid of AtMTP4, and the end was set about 40 amino acids after the Cation Efflux domain, corresponding to the last amino acid of ScZrg17. At the N terminus, with respect to the alignment, about 50% of the proteins started upstream from the first methionine of AtMTP4, with an average of 122 residues. At the C terminus, the alignment showed more conserved sequences and about 80% of proteins passed after the stop codon of Zrg17, with an average of 25 residues. Additional phylogenetic analysis using the Maximum Parsimony (MP) method (MEGA 3.1) was performed and gave congruent topologies confirming the confidence of the clustering (data not shown). Based on tree topology and confidence on nodes (bootstrap values >50), we defined 13 clusters containing characterised sequences (Figure 4), which were named by referring to one characterised member (usually the first identified). The ZnT9-like cluster contained murine and human ZnT9, previously called HUEL, that also have significant homology with a DNA-binding domain and a nuclear receptor interaction motif. ZnT9/HUEL was demonstrated to be predominantly localized to the cytoplasm and to translocate to the nucleus during the S phase of the cell cycle [34]. To avoid misleading labelling, the cluster containing the Mn^2+ ^transporter ShMTP1 was named MTP8 (AtMTP8 is the orthologue of ShMTP1 in *Arabidopsis thaliana*), following the nomenclature proposed for Arabidopsis at the PlantsT World Wide Web site [35]. Eleven of these 13 clusters clustered into three groups (Figure 3), which are detailed below. We hypothesized that proteins belonging to the same group may share the same metal specificity. Thus, for those transporters whose substrate is not known, the position within one of these groups may suggest the metal specificity.

#### Group I: zinc-CDFs

The first group included phyla specific members, such as the Zrc1-like, DmeF-like, ZitB-like, the ZnT1-like, and ZnT6-like clusters. The Zrc1-like cluster comprised only fungal CDFs originating from Ascomycetes, Basidiomycetes, and Zygomycetes. The bacterial specific DmeF-like and ZitB-like clusters included DmeF from *Cupriavidus *(synonym *Ralstonia*, or *Wautersia*) *metallidurans *CH34 which is a broad specificity transporter with a preference for Co^2+ ^and Zn^2+ ^[12], and the EcZitB protein from *E. coli*, which mainly transports Zn^2+ ^and Cd^2+ ^[6]. The ZitB-like cluster further clustered into two subclusters, one containing only members from Proteobacteria and the other only members from the Firmicute phylum. The ZnT1-like and ZnT6-like clusters comprised only metazoan sequences. Close to the ZitB-like cluster was a branch containing another Zn^2+ ^(Co^2+^, Cu^2+^, and Ni^2+^) transporter from *C. metallidurans*, RmCzcD, the first identified CDF member [10,29]. Additionally, this first group also included the Msc2-like and ZnT2-like clusters containing CDF members from fungi, metazoans and plants. The Msc2-like cluster included the 12-TMD members from plants, one for each of *A. thaliana, Populus trichocarpa *and *Oryza sativa*, the 12-TMD transporter Msc2 from *S. cerevisiae *and its homologs from Ascomycetes, Basidiomycetes, and Zygomycetes (one for each species), and the human 12-TMD HsZTL1 and the 15-TMD HsZnT5 transporters. The human Zn^2+ ^transporter HsZnT7 and its orthologue from the fly *Drosophila melanogaster *were also present in this cluster. Finally the ZnT2-like cluster contained plant members distributed in three subclusters, one sequence from the alga *Chlamydomonas reinhardtii*, one from Alveolata (*Cryptosporidium parvum*), one from the Zygomycete *Rhizopus oryzae*, and a subcluster of sequences from mammals and flies. Sequences belonging to the ZnT2-like and ZitB-like clusters, together with RmCzcD, corresponded to the CDF2 group described by Nies [4], while the DmeF-like and Msc2-like clusters matched with the CDF1 group [4]. Concerning the classification proposed by Gaither and Eide in 2001[3], the ZitB-like, Znt1-like, and MSC2-like clusters belonged to the CDF subfamily II and ZnT6-like, ZnT2-like, and Zrc1-like clusters to the CDF subfamily III [3].

Several CDF transporters belonging to Group I have been biochemically characterised; metal specificities have been mainly investigated by heterologous complementation, and also inferred from the phenotypes shown by gene-specific mutants, or over-expression of the transporter. Few transporters have been studied in reconstituted proteoliposomes or in everted membrane vesicles (Figure 4). Even though the substrate specificity shown in reconstituted proteoliposomes does not necessarily reflect the physiological substrate(s) *in vivo*, this technique is often used to study transporter kinetic mechanisms. In the ZnT2-like cluster, the plant transporters AtMTP1, AtMTP3, TgMTP1, and PtdMTP1 and the mammalian RnZnT2, MmZnT3, HsZnT3, and MmZnT4 [13,23,26,36-39] have been characterised. Other characterised members include the two orthologs RnZnT1 and MmZnT1 [40] from the Zn-T1-like cluster; HsZnT6 [41] from the ZnT6-like cluster; and ScMsc2, MmZnT5, HsZnT7, and HsZTL1 [19-21,42] from the Msc2-like cluster. Almost all these CDFs specifically transport Zn^2+^; only AtMTP1 displayed a low affinity for Cd^2+ ^in reconstituted proteoliposomes (100-fold lower than Zn^2+^) and TgMTP1 was shown to be able also to confer Ni^2+^, Cd^2+^, and Co^2+ ^tolerance when over-expressed in yeast [13,26]. The other characterised members of Group I display a broader specificity. For example, RmCzcD from *C. metallidurans *is able to bind Zn^2+^, Co^2+^, Cu^2+^, and Ni^2+ ^[29] and to alleviate Zn^2+^, Co^2+ ^and Cd^2+ ^toxicity when over-expressed in bacteria [10]; EcZitB, BsCzcD (ZitB-cluster), and DmeF are Zn^2+ ^and Co^2+^/Cd^2+ ^transporters, as measured in reconstituted proteoliposomes or in everted membrane vesicles [6,8,12]; the fungal ScZrc1, ScCot1, and SpZhf1 (Zrc1-like cluster) are Zn^2+ ^and Co^2+^/Cd^2+ ^transporters, as deduced by the gene-specific mutant phenotypes [43-45]. Hence CDF Group I contains characterised members that at least transport zinc (Figure 4). Other potential substrates are Co^2+^, Cd^2+^, and Ni^2+^, but neither Fe^2+ ^nor Mn^2+ ^are reported among the substrates. This group will be referred to as the Zn-CDF group.

#### Group II: iron/zinc-CDFs

This group includes the bacterial FieF-like and WmFieF-like clusters and the fungal MMT-like cluster (Figure 3). The MMT-like cluster comprises only fungal sequences from Ascomycetes, Basidiomycetes, and Zygomycetes (one for each species, with the exception of *S. cerevisiae*). MgMamB and sequences belonging to the FieF-like cluster were classified in the CDF3 group by Nies [4], and MMT-like and FieF-like clusters were classified in CDF subfamily I by Gaither and Eide [3]. Remarkably, the few characterised members of this group transport iron, among the other metals Zn^2+^, Co^2+^, Cd^2+^, Ni^2+^, but do not transport Mn^2+^. This is best illustrated by the bacterial iron transporters FieF from *E. coli *[7], MgMamB from *Magnetospirillum gryphiswaldense *[46], and by the mitochondrial yeast iron transporters ScMMT1 and ScMMT2 [47]. WmFieF from *C. metallidurans *is mainly involved in Fe^2+ ^detoxification, although it also mediates resistance against other metals such as Zn^2+^, Co^2+^, Cd^2+^, and Ni^2+^, as shown by overexpression in *E. coli *or in *C. metallidurans *metal sensitive mutants [12]. Site directed mutagenesis and isothermal titration calorimetry analyses in EcFieF demonstrated that Zn^2+^, Cd^2+^, and Fe^2+ ^are coordinated by four amino acids belonging to TMDs II (D45 and D49) and V (H153 and D157) [31]. This group will be referred to as the Fe/Zn-CDF group.

#### Group III: manganese-CDFs

This group comprises MTP8-like sequences (bootstrap value = 91), including the well characterised ShMTP1 and its paralogs ShMTP2, ShMTP3, and ShMTP4 from *S. hamata *[11], as well as the fungal PiMnT1 transporter from *Paxillus involutus *(D. Blaudez, M. Chalot, unpublished data) and the plant transporters AtMTP11 from *A. thaliana *and PtrMTP9, PtrMTP11.1, and PtrMTP11.2 from *P. trichocarpa *(B Montanini, D. Blaudez, M. Chalot, unpublished data and [48]). Within this group, the plant and fungal members clustered in specific subgroups and the fungal members further clustered into four subgroups (Figure 3). *Magnaporthe grisea*, *Neurospora crassa*, and *Stagonospora nodorum *had one transporter in each subgroup, whereas other fungi had one to three transporters, distributed in the four subgroups. The recent availability of annotated genomes from *Candida *species (Broad Institute [49]), allowed the identification of MTP8-like members also in yeast (one member for each *Candida *yeast genome clustered in the same subgroup). This distribution suggested that a MTP8-like transporter was lost in some yeasts (*S. cerevisiae*, for example) and that two duplication events probably occurred during the evolution of filamentous fungi. Finally, the MTP8-like cluster also contained three CDFs from the worm *Caenorhabditis elegans*, three from the alga *C. reinhardtii*, and two from the Entamoebidae *Entamoeba histolytica*. Mammalian and prokaryotic CDFs were not found within this cluster; indeed BLASTP searches against complete mammalian or prokaryotic genomic databases, using MTP8-like sequences as query, did not match with any sequence. Sequences belonging to the MTP8 cluster may have originated from a common eukaryotic ancestor and spread in metazoa (*C. elegans*), fungi, algae, plants, and protists (*E. histolytica*). Further duplication events in different phyla generated the plant and fungal subgroups, while some phyla (e.g. mammals) apparently lost this type of CDF (Figure 3). Most interestingly, the nine characterised CDFs were able to complement specifically the growth defect of the yeast strain *pmr1*Δ on high manganese concentration, and did not support growth of other metal-hypersensitive strains, thus suggesting that they are specifically involved in manganese homeostasis or tolerance ([11,48], Figure 4 and unpublished data). This group will be referred to as the Mn-CDF group.

Additionally, the human ZnT9 protein clustered with plant, algal and bacterial CDF members in the ZnT9-like cluster, and this was thus the only mixed prokaryotic-eukaryotic CDF cluster (Figure 3). BLASTP analysis using ZnT9-like sequences as query showed that this CDF cluster was present in other mammalian genomes (mouse, orang-utan, and dog), in other metazoans, in plants, in Archaea and in Eubacteria. Mammalian Znt9 proteins were classified in CDF subfamily I by Gaither and Eide [3]. The distant Zrg17 cluster contained only members from Ascomycetes, implying that this CDF cluster was specific to this class of fungi. Indeed, BLASTP analyses against public databases using Zrg17-like sequences as a query retrieved sequences only from Ascomycetes.

### Features related to group specificity

We further searched for residues that could represent candidate sites of functional divergence of the CDF groups. Remarkably, Mn-CDF sequences could be differentiated by the consensus sequence DxxxD (x = any amino acid) in TMD V, which appears as HxxxD in all other CDF sequences from the Zn- and Fe/Zn-CDFs, D being the highly conserved aspartate residue important for CDF function ([14,29,30] and Table 1). In Zrg17-like and in ZnT9-like clusters, sequences did not match with any of these patterns, since TxxxS/T and VxxxD patterns were found in the same topological positions, respectively (Figure 4). When the TMD V region was excluded from the alignment, the tree topology and bootstrap values did not change appreciably, suggesting that the close phylogenetic relationships were not only based on TMD V amino acid conservation. Since the group-conserved H/D residue is four residues distant the highly conserved aspartate within TMD V, the two residues will interact on the same face of the α-helix. These residues may play important but different roles in the CDF groups. In EcFieF the corresponding residue, H153, is one of the four amino acids that directly coordinates ions during binding and transport and it faces the cytosolic side of the membrane [31]. The cysteine thiolate, histidine imidazole, and aspartate/glutamate carboxylate have similar properties as ligand groups for Fe^2+ ^and Zn^2+ ^metals, while Mn^2+^, like other hard metals, prefers carboxylate toward N-imidazole ligand group [50]. In the Zn-CDF PtdMTP1 the corresponding histidine located on TMD V was changed to aspartate. The H260D substitution in PtdMTP1 led to the loss of any detectable function in Zn^2+ ^homeostasis, without changing the protein localisation, demonstrating that these aspartate and histidine residues do not have the same role (Figure 2). However the H260D variant of PtdMTP1 was not able to complement the manganese-hypersensitive strain *pmr1*Δ.

In TMD II a histidine (Zn-CDF)/aspartate (Mn-CDF) residue is conserved within each specific group, although in the Fe/Zn-CDF group, both aspartate (FieF-like cluster) and histidine (MMT-like and WmFieF-like clusters) are present (Figure 4). These residues reside four amino acids upstream from the highly conserved aspartate in TMD II and in EcFieF they correspond to D45, one of the four metal coordination residues that is located close to the non cytosolic side of the membrane [31].

Another key structural feature is the presence of a histidine-rich region in most CDFs, except the Mn-CDF, the ZnT9-like, FieF-like, and WmFieF-like transporters (Figure 4). Most Zn-CDF transporters usually display histidine-rich (or histidine/serine-rich) regions at N, C termini in and/or between TMDs IV and V. The function of the histidine-rich loop was demonstrated in AtMTP1, where the mutated version lacking the histidine-rich loop was unable to bind Zn^2+ ^[26], while in EcZitB neither the N-terminal nor the C-terminal regions were required for function [30]. In ZnT6-like sequences the histidine-rich loop is substituted by a serine-rich loop and a histidine/serine-rich region is also present at the C-terminus. In ZnT1-like transporters extra cysteine/serine rich regions are found in the TMDs V-VI loop and at the C terminus.

Mn-CDF members present a serine-rich region at the N terminal. Sequences belonging to Zrg17-like and MMT-like clusters also have a histidine/serine-rich loop, although located between TMDs III and IV (Figure 4).

A specific feature of the Zn-CDFs is the presence of few cysteine residues conserved within some clusters: sulfhydryl moieties are known to have a role in metal binding (mostly Zn^2+ ^and Cd^2+^). In the ZnT1-like cluster five cysteine residues are conserved at the cytosolic C terminus; another cysteine residue is conserved in TMD III in both ZnT1-like and Zrc1-like clusters; the Msc2-like cluster shows a cysteine residue conserved in all sequences at TMD III (by referring to the CDF domain). Other cysteine residues are conserved in the ZnT2-like cluster: in order to test the importance of these cysteine residues for function and their conservation during evolution, all five cysteine residues (C30, C35, C64, C291, and C357) were changed to serine in the poplar PtdMTP1 member. C30, C64, and C291 were conserved within the ZnT2-like cluster, and C35 and C357 were conserved only in the plant members of this cluster. Substitution of the C35 residue abolished the function of the transporter, while the C30S and C64S substitutions only partially affected PtdMTP1 function (Figure 2). The C291S and C357S substitutions did not have any effect on Zn^2+ ^tolerance: the expression of these *PtdMTP1 *mutated versions allowed growth of the Zn-yeast hypersensitive strain *zrc1*Δ up to 20 mM ZnCl_2 _(Figure 2, Table 1, and data not shown). All the mutant proteins still localised on the tonoplast, as shown by C terminal chimeric fusion with GFP. To gain insights into the role of cysteine residue in the oligomerisation of PtdMTP1, Western blot analyses were performed with the mutant proteins as previously described [23]. All the mutant proteins still formed homo-oligomers (data not shown). The Fe/Zn-CDF group did not have any conserved cysteine residue, while the Mn-CDF group displayed a highly conserved cysteine at the cytosolic side of TMD IV.

## Discussion

### A modified signature for the CDF family

In the present work, we propose a modified signature that better takes into account the newly characterised CDF transporters. Retrieved at Prosite [33] with the proposed modified CDF signature, the 930 hypothetical CDFs still mostly clustered in the three groups, thus suggesting that a good representative sample of CDF sequences was originally used for analysis. It is possible also to suggest their substrate specificity: 268 were classified in the Fe/Zn-CDF group, 561 in the Zn-CDF group, and 39 in the Mn-CDF group. Few of the sequences originally selected for the protein alignment used to construct the signature were not retrieved by the new signature, since the three databases available at Prosite did not include some data from genome sequencing projects. Some amino acid residues along the signature were specifically present only in a few clusters: this is the case of the amino acid at the position 10 (H89 in PtdMTP1, Figure 1) that is always an aspartate in the Mn-CDF group and in the FieF-like cluster, while a histidine residue is present in the same position in the Zn-CDF group and in MMT-like, WmFieF-like, and ZnT9-like clusters. At position 14 (D93 in PtdMTP1, Figure 1) aspartate is the most frequent amino acid, although some sequences belonging to the Proteobacteria phylum displayed a histidine residue in that position. These two positions, along with the fully conserved glycine were the most critical signature residues, since when changed to other amino acids many non-CDF sequences were retrieved. These amino acids are essential for function, as confirmed by mutagenesis studies (Table 1 and Figure 2), suggesting that the conservation of these amino acids has been maintained by evolutionary pressure.

### Evolutionary relationships in the CDF family

CDF member distribution in the phylogenetic tree was not related to organism taxonomy, but rather to substrate specificity suggesting ancient duplication events followed by subfunctionalization (metal specialization) in a common ancestor to prokaryotes and eukaryotes. More recent duplications or gene losses could have arisen in several taxa and lineages leading to a complex distribution pattern of CDF isoforms (orthologous and paralogous copies) in living organisms. Based on amino acid sequence similarities and phylogeny of a set of 273 CDF sequences, and on biochemical features of some well-characterised CDF members, the CDF family could be divided into three major groups, one containing Zn-CDF transporters (ZnT2-like, ZnT1-like, ZnT6-like, Zrc1-like, Msc2-like, ZitB-like, and DmeF-like clusters), another enclosing Fe/Zn-CDF transporters (MMT-like, FieF-like, and WmFieF-like clusters) and a third containing only Mn-CDF transporters (MTP8-like sequences) (Figure 3 and Figure 4).

An update of CDF classification is therefore suggested in order to take into better account phylogenetic and functional features. Three groups are proposed: Zn-CDF, Fe/Zn-CDF and Mn-CDF. Ten of the 11 clusters belonging to these groups were already classified in CDF subfamilies by Nies [4] or by Gaither and Eide [3]. In the particular Zn-CDF group contained CDF groups 1 and 2 [4] and CDF subfamilies II and III [3], while Fe/Zn-CDF group contained CDF group 3 [4] and CDF subfamily I [3]. MTP8-like clusters was not present in those classifications because members belonging to this cluster had not then been characterised. The Zrg17-like cluster was very distant from the Zn-CDF group and displayed only a partial signature. It was therefore not included in the Zn-CDF group, even though it had similar biochemical characteristics. The ZnT9-like cluster (previously classified in CDF subfamily I [3]) was found to be close to the Mn-CDF group, but the lack of biochemical characterisation and a weak bootstrap value (23) prompted us to exclude this cluster from the Mn-CDF group (Figure 3 and Figure 4). Sequences belonging to the ZnT9-like cluster might have an ancient origin from a common ancestor and spread in bacteria, metazoans, algae, and plants. Its absence in fungal genomes suggests that this type of CDF has been lost in fungi. The lack of conserved residues in the active site and the potential DNA binding properties of the mammalian ZnT9 proteins [34] suggest additional or different roles of these proteins that nonetheless retained high similarity with the CDF family.

The Mn-, the Zn- and Fe/Zn-CDF groups share many conserved residues, suggesting that they derived from a common ancestor. Newly identified CDF members can be assigned to one of these groups and therefore their substrate specificity can be inferred: for example in plants, only members belonging to the Zn-CDF [13,23,26,36] or to the Mn-CDF [11,48] groups have been functionally characterised. The position of AtMTP6 and orthologous proteins in the Fe/Zn-CDF group suggests a Fe^2+ ^selectivity for these CDF members.

The number of transmembrane domains predicted in each transporter is roughly conserved in each cluster, most including from 4 to 6 TMDs, depending on the method used for the prediction. Notably, the Msc2-like cluster included all members with a relatively high TMD number (12 TMDs in Msc2, HsZTL1, and AtMTP12, to 15 TMDs in HsZnT5).

Subcellular localisation of CDF transporters is not always conserved within clusters. The Fe/Zn-CDF group mainly contained sequences from prokaryotes (Archaea or Eubacteria) and fungi. The mitochondrial localisation of ScMMT1 and ScMMT2 [47] corresponds to the plasma membrane location of the prokaryotic members [7,12]. Within the ZnT2-like cluster, however, plant members localised either on the tonoplast (AtMTP1 [51], PtdMTP1 [23]) or on the plasma membrane (TgMTP1, [52]), while mammalian CDF transporters were found on vesicles [37-39]. The vacuolar membrane located transporters Zrc1 and Cot1 from *S. cerevisiae *clustered together with the reticulum endoplasmic localised Zhf1 from *Schizosaccharomyces pombe *[45].

The relatively high number of Zn-CDFs found in databases compared with the number of Mn-CDFs may reflect the evolutionary pressure to which the organisms were subjected. Indeed some Zn-CDFs display a broad specificity, which gives a potentially useful trait to the organism. The only CDF transporter known to transport Ni^2+ ^(over Zn^2+^) is present in the Ni-hyperaccumulator species *Thlaspi goesingense *[13]. Similarly DmeF, RmCzcD, and WmFieF displayed a broad specificity [5,12], and *C. metallidurans *has been frequently found in sediments and soils with a high content of various heavy metals from diverse geographical locations. The need for a Mn^2+ ^detoxification (or homeostasis) system driven by CDF transporters could have represented a less important pressure during evolution or could act in parallel with other transport systems (for example the Ca^2+^/Mn^2+ ^P-type ATPase Pmr1 in fungi and ECA1 in plants or the cation exchanger CAX2 in plants [53]).

### Functional analyses of the eukaryotic Zn-CDF PtdMTP1

In the present study we identified residues that are crucial for function in the model eukaryotic Zn-CDF PtdMTP1. Amino acid substitutions of conserved aspartate and histidine residues in TMDs II and V led to the loss of almost any detectable function in complementation tests. In EcFieF four amino acids belonging to TMD II (D45 and D49) and to TMD V (H153 and D159) are the metal coordination residues that directly interact with the substrate during binding and transport [31]. The corresponding amino acids are likely to have the same role in other CDF transporters, since at least in EcZitB, RmCzcD and PtdMTP1, substitutions in these residues completely abolish the function (Table 1).

Concerning PtdMTP1, all the mutant proteins were expressed and localised using GFP to the tonoplast, as found for the wild type protein [23], with the exception of the mutant D288A/E proteins that localised on vesicle-like structures (data not shown). Thus for the substitutions D288A/E (TMD VI) we cannot conclude whether the loss of function was due to the mis-localisation or to a reduced activity of the transporter. Additionally the D181E substitution in the corresponding aspartate residue in the Zn^2+^/Co^2+ ^bacterial transporter RmCzcD prevented the correct membrane localisation and induced a partial degradation of the mutant protein [29]. Therefore this aspartate residue appears to be critical for CDF protein stability and localisation in organisms from different kingdoms. Likewise in the yeast Pmr1, a Golgi-localised Ca^2+^/Mn^2+ ^P-ATPase transporter, the substitutions D778E and D856E in TMD VI and TMD VIII were responsible for ER retention [54]. The G118A substitution abolished function as well. The topological position of G118 (upstream from TMD III), its unique conservation pattern among the CDF transporters and its significance in protein function (at least for PtdMTP1) suggests a role in the formation of a correct protein structure. Regarding substitutions of cysteine residues, only the C35S substitution dramatically affected protein function. The four other substitutions resulted in a reduction of PtdMTP1-mediated zinc resistance (C30S and C64S) or did not affect protein function (C291S and C375S). Based on its functional role and its conservation in a well defined phylogenetic group (plant members of the ZnT2-like cluster), we can hypothesise that the C35 residue has been positively selected throughout plant evolution and has contributed to adaptation. The possible role of these amino acids in protein oligomerisation was investigated in this work by Western blot analysis. Although previous results in the presence of DTT suggested an oligomerisation mediated by disulfide bridges [23], the variant proteins still form oligomeric structures, suggesting that the cysteine residues are dispensable in forming the quaternary structure of PtdMTP1 Zn^2+ ^transporter (data not shown). The conserved cysteine residues, located in the N and C termini, are likely to be involved in substrate binding rather than substrate transport. The occurrence of more than one cysteine residue might be an indication of redundant function and the presence of double-substituted cysteine residues might be necessary to abolish protein function and/or oligomerisation.

### Molecular features related to metal specificity

The identification of molecular determinants that may reflect metal specificity is an interesting research field with application to bioremediation. Several features may contribute to metal specificity in CDF members, nevertheless the widely conserved aspartate residues in TMDs II and V cannot alone be responsible for metal specificity, because they are conserved in transporters with different metal specificities. The mechanism of metal selectivity might be guaranteed by the coordination chemistry in the chemical context of the immediate binding site neighbourhood. Zn^2+ ^usually favours a tetrahedral coordination geometry. Mn^2+ ^and Fe^2+ ^are mainly found in octahedral coordination even though a tetrahedral coordination geometry of ligands is also favoured by these metals. The amino acids located in TMD V, four residues upstream from the highly conserved aspartate, seemed to be candidates of choice for determination of metal specificity of the Zn- and Fe/Zn-CDFs (histidine) or of the Mn-CDFs (aspartate). At least four features suggested that these residues are likely to be important in the determination of substrate specificity: (i) they were not interchangeable, at least in the eukaryotic Zn^2+ ^transporter PtdMTP1 (Figure 2), (ii) they were specifically conserved within groups (Figure 4), (iii) they resided in close contact with the highly conserved and functionally important aspartate, on the polar side of the amphipathic helix V, (iv) they faced the cytosolic side of the helix, where they can contact the substrate. These amino acids correspond to H153 in EcFieF, which is the closest coordination residue to the cytoplasm, *cis *to metal uptake [31]. The H260D variant of PtdMTP1 was not able to complement the Mn-hypersensitive strain *pmr1Δ*. We can thus infer that this histidine residue is necessary for PdtMTP1-mediated resistance on Zn^2+^, but we cannot speculate whether the presence of aspartate, instead of histidine, is sufficient for manganese transport.

The other conserved and amphipathic helices did not contain any completely group-conserved residue that may be related to metal specificity. Nevertheless this does not exclude their involvement in conferring metal specificity. Even if we cannot conclude that the presence of this distinctive amino acid (D or H) may be sufficient to discriminate the substrate, this could be a starting point for further investigations. Other mechanisms, such as the interactions with metal chelating proteins or cofactors and the involvement of histidine rich regions (Figure 4), have to be taken into account in the first step of metal binding. Nevertheless the Fe/Zn-CDFs FieF from *E. coli *and *C. metallidurans *do not have any histidine rich-loops [7,12] (Figure 4). Furthermore, some CDF transporters have been shown to be active in reconstituted proteoliposomes [6,7,26], demonstrating that at least these transporters did not require any interaction with other proteins or cofactors to sustain metal transport.

## Conclusion

In the present study phylogenetic and functional analyses have allowed the identification of three major substrate-specific CDF groups: Zn-, Fe/Zn-, and Mn-CDF. A modified signature that better fits with the increased number of hypothetical CDF transporters is described and might be useful to identify new CDF transporters. The metal specificity of newly identified CDF transporters can be then deduced by their classification into one of these three groups, but only an exhaustive functional characterization of CDF members will allow us to confirm this hypothesis. In particular the role of the group-conserved aspartate/histidine residue in metal selectivity has to be verified by complementation tests in other metal-specific sensitive strains. Moreover site directed mutagenesis analyses on other regions potentially involved in substrate specificity and in transporters belonging to the different CDF groups will be useful in finding the molecular determinants of metal selectivity of the CDF family.

## Methods

### Sequence and phylogenetic analyses

Deduced amino acid sequences, belonging to characterised or hypothetical CDF transporters, have been retrieved from Genebank and SwissProt and from a set of annotated genomes. These include the Archaea *Methanosarcina acetivorans *and *Methanosarcina mazei*, the actinobacteria *Corynebacterium glutamicum *and *Streptomyces coelicolor*, the cyanobacterium *Nostoc punctiforme*, the firmicutes *Bacillus cereus*, *Bacillus subtilis*, and *Clostridium tetani*, the proteobacteria *Agrobacterium tumefaciens*, *E. coli*, *Thiomicrospira crunogena*, and *C. metallidurans*, the alga *C. reinhardtii*, the dicotyledonous plants *A. thaliana *and *P. trichocarpa *and the monocotyledonous plant *O. sativa*, the protist *E. histolytica*, the Ascomycetes *Ashbya gossypii *(hemiascomycete), *Aspergillus fumigatus*, *Aspergillus nidulans*, *Botrytis cinerea*, *Candida albicans*, *Fusarium graminearum*, *M. grisea*, *N. crassa*, *S. cerevisiae*, *Sclerotinia sclerotiorum*, and *S. nodorum*, the Basidiomycetes *Phanerochaete chrysosporium *and *Ustilago maydis*, the Zygomycete *R. oryzae*; the nematode *C. elegans*, the insect *D. melanogaster*, and the mammal *Homo sapiens*. Sequences homologous to Zrg17 from *S. cerevisiae *were retrieved by BLASTP search in the above-described genomes, since these members were not annotated. Sequences that displayed significant truncation have been discarded. CDF amino acid sequences (see accession numbers in additional file 1) were aligned using Gonnet as protein weight matrix, available in MEGA (version 3.1) [55]. Molecular evolutionary analyses were conducted using MEGA (version 3.1) [32]: for the construction of the phylogenetic tree the Maximum Parsimony (MP) or the Neighbor-Joining (NJ) method and the bootstrap (1000 replicates) phylogeny test were applied on the multiple sequence alignment. Sequence distances were calculated with the Poisson correction model with uniform rates among sites.

Logo representation of the CDF signature was constructed with the web interface program WebLogo [56,57] on the basis of the alignment of CDF members listed in additional file 1, with the exception of the Zrg17-like sequences. For TMD II and V representation, alignments corresponding to each individual cluster or group were used as queries. The signature was verified at ScanProsite [33] against all the three available databases (Swiss-Prot, TrEMBL and PDB Databases), with default settings for pattern options.

### Yeast strains, media, and transformation

The yeast strains used for the heterologous expression of *PtdMTP1 *were BY4741 (MATa *his3*Δ*1 leu2*Δ*0 met15*Δ*0 ura3*Δ*0*), *zrc1*Δ (MATa *his3*Δ*1 leu2*Δ*0 met15*Δ*0 ura3*Δ*0 ZRC1*::*kanMX4*), and *pmr1*Δ (MATa *his3*Δ*1 leu2*Δ*0 met15*Δ*0 ura3*Δ*0 PMR1*::*kanMX4*). The mutants used derived from the parental strain BY4741 and were all obtained from Euroscarf [58]. Growth was in YPD or in synthetic defined (SD) medium with 2% (w/v) galactose, supplemented as necessary [59]. Yeast cells were transformed as described elsewhere [60]. For metal tolerance assays, yeast was grown in liquid SD medium (with 2% [w/v] galactose) at 30°C until O.D. _600 nm _= 1, and drop assays were performed on SD plates containing different concentrations of Zn^2+ ^or Mn^2+^.

### Site-directed mutagenesis

Mutant *PtdMTP1 *plasmid clones were generated by oligonucleotide directed PCR mutagenesis, using pYES2-*PtdMTP1 *as a template for the PCR (Table 2). The mutated versions of *PtdMTP1 *were cloned in pYES2 and expressed in the Zn-hypersensitive strain *zrc1*Δ. Cloning, site-directed mutagenesis, construction of the expression plasmid pYES2-GFP, and Western blotting analysis were described previously [23]. The presence of targeted mutations in all plasmid constructs was verified by DNA sequencing.

## Abbreviations

TMD, Transmembrane domain; CDF, Cation Diffusion Facilitator; GFP, Green Florescence Protein; MTP, Metal Tolerance Protein; ZnT, Zinc Transporter; SLC30, Solute carrier family 30.

## Authors' contributions

BM carried out the phylogenetic analysis, participated to the site-directed mutagenesis studies and drafted the manuscript, DB participated in the site-directed mutagenesis studies and in the design of the study, SJ helped in the conception and interpretation of the phylogenetic analysis, DS and MC participated in the design of the study, and revised the manuscript critically. All the authors have read and approved the final manuscript.

## Supplementary Material

Additional file 1 The protein ID of the CDF amino acid sequences used for phylogenetic analysis is reported, with the taxonomy classification, accession numbers, and the related database. For sequences retrieved from genome sequencing projects the corresponding database source is reported. Click here for file

## Figures and Tables

**Table 1:** Mutagenesis studies on CDF members

	**Prokaryotic members**						**Eukaryotic member**	
	**RmCzcD**		**EcZitB**		**EcFieF**		**PtdMTP1**	

Topological position	AA substitution	Phenotype	AA substitution	Phenotype	AA substitution	Phenotype	AA substitution	Phenotype

N ter							C30S	Partially active^e^
N ter							C35S	No function^e^
N ter							C64S	Partially active^e^
N ter	H5R	Partially active^a^						
N ter	H7R	No function^a^						
N ter	H9R	Partially active^a^						
TMD I	E31D	Partially active^a^	E35D	Partially active^b^				
TMD I			E35A	Partially active^b^				
TMD I	E31K	No function^a^						
TMD II			D50E	Partially active^a^				
TMD II			D50A	No function^a^			D86A	No function^f^
**TMD II**					**D45C**	**Wild type**^c^		
**TMD II**	**H49R**	**Not expressed**^a^	**H53R**	**No function**^a^			**H89K**	**No function**^f^
**TMD II**	**H49A**	**No function**^a^					**H89A**	**No function**^f^
TMD II	M5OL	Hypersensitive^a^	M54L	Partially active^a^				
**TMD II**					**D49C**	**Wild type**^c^		
**TMD II**	**D53E**	**Hypersensitive**^a^						
**TMD II**	**D53A**	**Hypersensitive**^a^	**D57A**	**No function**^a^	**D49A**	**No function**^c^	**D93A**	**No function**^f^
TMD II	D53N	Hypersensitive^a^						
TMDs II-III loop							G118A	No function^e^
TMD IV			N135A	No function^a^				
**TMD V**					**H153C**	**Partially active**^c^		
**TMD V**	**E154D**	**No function**^a^	**H159E**	**No function**^a^			**H260D**	**No function**^e^
**TMD V**	**E154N**	**No function**^a^	**H159R**	**No function**^a^				
**TMD V**					**D157C**	**Partially active**^d^		
**TMD V**	**D158E**	**No function**^a^	**D163E**	**No function**^a^			**D264E**	**No function**^e^
**TMD V**	**D158A**	**No function**^a^	**D163A**	**No function**^a^	**D157A**	**No function**^d^	**D264A**	**No function**^e^
TMD V			S167A	No function^a^				
			W182L	Partially active^a^				
TMD VI	D181A	Partially active^a^	D186A	No function^a^			D288A	No function^e^
TMD VI	D181E	Hypersensitive^a^	D186E	Partially active^a^			D288E	No function^e^
TMD VI	D181N	Hypersensitive^a^						
C ter							C291S	Wild type^e^
C ter			E214A	No function^a^				
C ter	H237R	No function^a^						
C ter	H251A	Partially active^a^						
C ter			W245L	Partially active^a^				
C ter			G248T	Partially active^b^				
C ter							C357S	Wild type^e^
C ter	H280A	No function^a^						
C ter	H298A	No function^a^						
C ter	C290S	Hypersensitive^a^	C294S	Wild type^a^				

The results of site-directed mutagenesis analyses carried out on four CDF transporters ([14, 23, 29-31] and present study) are reported with the corresponding topological position. RmCzcD: Zn-CDF from *C. metallidurans*; EcZitB: Zn-CDF from *E. coli*; EcFieF: Fe/Zn-CDF from *E. coli*; PtdMTP1: Zn-CDF from *P. trichocarpa *× *deltoides*. "no function": the amino acid substitution led to the loss of any detectable function in the complementation test, comparable with the empty vector; "hypersensitive": the amino acid substitution resulted in a more sensitive phenotype, compared with the empty vector-transformed cells; "partially active": the phenotype is intermediate between the empty vector- and the wild type gene-transformed cells. EcFieF metal coordination residues [31] and the corresponding amino acids in the other transporters are in bold. References: ^a^, [29]; ^b^, [30]; ^c^, [31]; ^d^, [14]; ^e^, this study; ^f^, [23].

**Table 2:** Oligonucleotides used for the introduction of amino acid substitutions in PtdMTP1

Primer name	Position	Sequence	Substitution
C30Sf	72-102	TGGAGGAAGCAAGGGAaGCGGGGAAGCACCT	C30S
C30Sr	102-72	AGGTGCTTCCCCGCtTCCCTTGCTTCCTCCA	C30S
C35Sf	87-118	TGCGGGGAAGCACCTTgTGGATTTTCAGAT	C35S
C35Sr	118-87	ATCTGAAAATCCAcAAGGTGCTTCCCCGCA	C35S
C64Sf	175-205	ATATCCGTGGCACTTTcTATAGTCTTCATG	C64S
C64Sr	205-175	CATGAAGACTATAgAAAGTGCCACGGATAT	C64S
G118Af	343-372	CAATCTTATGcATTTTTTAGGATTGAGATT	G118A
G118Ar	372-343	AATCTCAATCCTAAAAAATgCATAAGATTG	G118A
H260Df	764-793	AAGGAGCTTATCTCgATGTACTTGGGGATT	H260D
H260Dr	793-764	AATCCCCAAGTACATcGAGATAAGCTCCTT	H260D
D264Ef	775-804	CTCCATGTACTTGGGGAaTCCATCCAGAGT	D264E
D264Er	804-775	ACTCTGGATGGAtTCCCCAAGTACATGGAG	D264E
D264Af	775-804	CTCCATGTACTTGGGGcTTCCATCCAGAGT	D264A
D264Ar	804-775	ACTCTGGATGGAAgCCCCAAGTACATGGAG	D264A
D288Ef	847-876	GAGTGGAAGATAATTGAaCTGATCTGCACC	D288Ef
D288Er	876-847	GGTGCAGATCAGtTCAATTATCTTCCACTC	D288Ef
D288Af	847-876	GAGTGGAAGATAATTGcTCTGATCTGCACC	D288A
D288Ar	876-847	GGTGCAGATCAGAgCAATTATCTTCCACTC	D288A
C291Sf	855-885	ATAATTGATCTGATCTcCACCCTAATCTTT	C291S
C291Sr	885-855	AAAGATTAGGGTGgAGATCAGATCAATTAT	C291S
C357Sf	1053-1083	AAGATTCTCTTGGCTTcTCATGTGAAAATC	C357S
C357Sr	1083-1053	GATTTTCACATGAgAAGCCAAGAGAATCTT	C357S

For each oligonucleotide the position on the *PtdMTP1 *coding sequence is shown, together with the sequence and the target mutation. Nucleotides written in lower case were mutated with respect to the original sequence.
