# Supplementary material for: Phylogenetic and functional analysis of the Cation Diffusion Facilitator (CDF) family: improved signature and prediction of substrate specificity
Source: BMC Genomics. 2007 Apr 23;8:107. doi: 10.1186/1471-2164-8-107 (PMC1868760; doi:10.1186/1471-2164-8-107)
Supplement: Additional file 1 — The protein ID of the CDF amino acid sequences used for phylogenetic analysis is reported, with the taxonomy classification, accession numbers, and the related database. For sequences retrieved from genome sequencing projects the corresponding database source is reported. [file 1471-2164-8-107-S1.doc]

| **Taxonomy** | | |  | **Protein ID** | **Accession number references** | | |
| --- | --- | --- | --- | --- | --- | --- | --- |
| **Kingdom** |  | **Phylum** | **Species** |  | **Accession number** | **Accession number Database** | **Genome Database** |
| **Archaea** |  |  |  |  |  |  |  |
|  |  | Crenarchaeota | *Pyrobaculum aerophilum str. IM2* | PAE2715 | NP_560205.1 | Genebank |  |
|  |  |  | *Sulfolobus solfataricus P2* | SulfsCzcD | AAK42415.1 | Genebank |  |
|  |  | Euryarchaeota | *Methanocaldococcus jannaschii* | Mj0449 | Q57891 | Swiss Prot |  |
|  |  |  | *Methanosarcina acetivorans str. C2A* | MA0549 | AAM03993.1 | Genebank | Broad Institute |
|  |  |  | *Methanosarcina acetivorans str. C2A* | MA0617 | AAM04061.1 | Genebank | Broad Institute |
|  |  |  | *Methanosarcina acetivorans str. C2A* | MA0805 | AAM04244.1 | Genebank | Broad Institute |
|  |  |  | *Methanosarcina acetivorans str. C2A* | MA1117 | AAM04538.1 | Genebank | Broad Institute |
|  |  |  | *Methanosarcina acetivorans str. C2A* | MA2085 | AAM05485.1 | Genebank | Broad Institute |
|  |  |  | *Methanosarcina acetivorans str. C2A* | MA3366 | AAM06735.1 | Genebank | Broad Institute |
|  |  |  | *Methanosarcina acetivorans str. C2A* | MA4005 | AAM07355.1 | Genebank | Broad Institute |
|  |  |  | *Methanosarcina acetivorans str. C2A* | MA4394 | AAM07736.1 | Genebank | Broad Institute |
|  |  |  | *Methanosarcina mazei Go1* | MM0167 | AAM29863.1 | Genebank | University of Goettingen |
|  |  |  | *Methanosarcina mazei Go1* | MM1076 | AAM30772.1 | Genebank | University of Goettingen |
|  |  |  | *Methanosarcina mazei Go1* | MM1711 | AAM31407.1 | Genebank | University of Goettingen |
|  |  |  | *Methanosarcina mazei Go1* | MM1778 | AAM31474.1 | Genebank | University of Goettingen |
|  |  |  | *Methanosarcina mazei Go1* | MM2778 | AAM32474.1 | Genebank | University of Goettingen |
|  |  |  | *Pyrococcus horikoshii OT3* | PhCzcD | NP_142822.1 | Genebank |  |
| **Eubacteria** |  |  |  |  |  |  |  |
|  |  | Actinobacteria | *Arthrobacter aurescens* | AaCzcD | AAS20069.1 | Genebank |  |
|  |  |  | *Arthrobacter aurescens* | AAS20133.1 | AAS20133.1 | Genebank |  |
|  |  |  | *Corynebacterium glutamicum* | Cgl1281 | BAB98674.1 | Genebank | Kitasato Univ. |
|  |  |  | *Corynebacterium glutamicum* | Cgl2783 | BAC00177.1 | Genebank | Kitasato Univ. |
|  |  |  | *Streptomyces coelicolor A32* | SCO0776 | CAC14341.1 | Genebank | Sanger Institute |
|  |  |  | *Streptomyces coelicolor A32* | SCO1310 | CAC42857.1 | Genebank | Sanger Institute |
|  |  |  | *Streptomyces coelicolor A32* | SCO2772 | CAB87209.1 | Genebank | Sanger Institute |
|  |  |  | *Streptomyces coelicolor A32* | SCO3024 | CAB88908.1 | Genebank | Sanger Institute |
|  |  | Aquificae | *Aquifex aeolicus* | AQ_2073 | O67851 | Swiss Prot |  |
|  |  | Cyanobacteria | *Nostoc punctiforme* | NpF0707 | ZP_00109054 | Genebank | DOE Joint Genome Institute |
|  |  |  | *Nostoc punctiforme* | NpF1794 | ZP_00109098 | Genebank | DOE Joint Genome Institute |
|  |  |  | *Nostoc punctiforme* | NpF2455 | ZP_00110704 | Genebank | DOE Joint Genome Institute |
|  |  |  | *Nostoc punctiforme* | NpR3104 | ZP_00107840 | Genebank | DOE Joint Genome Institute |
|  |  |  | *Synechococcus elongatus PCC 7942* | Synpcc7942_1989 | YP_401006.1 | Genebank |  |
|  |  |  | *Synechococcus sp. CC9605* | Syncc9605_1040 | YP_381353.1 | Genebank |  |
|  |  |  | *Synechocystis sp. PCC 6803* | sll1263 | P74068 | Swiss Prot |  |
|  |  | Deinococcus-Thermus | *Deinococcus radiodurans* | DrDR1236 | Q9RUZ4 | Swiss Prot |  |
|  |  |  | *Thermus thermophilus* | TtCzrB | CAC83722.1 | Genebank |  |
|  |  | Firmicutes | *Bacillus cereus ATCC 14579* | BC_1701 | AAP08677.1 | Genebank | INRA |
|  |  |  | *Bacillus cereus ATCC 14579* | BC_2261 | AAP09225.1 | Genebank | INRA |
|  |  |  | *Bacillus cereus ATCC 14579* | BC_4404 | AAP11317.1 | Genebank | INRA |
|  |  |  | *Bacillus subtilis* | BsCzcD | NP_390542.1 | Genebank | BSNR |
|  |  |  | *Bacillus subtilis* | BsYeaB | P46348 | Swiss Prot | BSNR |
|  |  |  | *Clostridium tetani E88* | CTC_01013 | AAO35599.1 | Genebank | University of Goettingen |
|  |  |  | *Clostridium tetani E88* | CTC_02458 | AAO36923.1 | Genebank | University of Goettingen |
|  |  |  | *Enterococcus faecalis* | EfEF0859 | Q837I0 | Swiss Prot |  |
|  |  |  | *Staphylococcus aureus* | StaCzcD | BAA36686.1 | Genebank |  |
|  |  |  | *Staphylococcus aureus* | StaRzcB | AAC32485.1 | Genebank |  |
|  |  |  | *Streptococcus agalactiae A909* | SAK_1232 | YP_329849.1 | Genebank |  |
|  |  |  | *Streptococcus agalactiae A910* | SAK_0514 | ABA44896.1 | Genebank |  |
|  |  |  | *Streptococcus pneumoniae R6* | spr1672 | AAL00475.1 | Genebank |  |
|  |  | Proteobacteria | *Agrobacterium tumefaciens* | Atu0891 | NP_531589.1 | Genebank | NCBI |
|  |  |  | *Agrobacterium tumefaciens* | Atu0991 | NP_531689.1 | Genebank | NCBI |
|  |  |  | *Agrobacterium tumefaciens* | Atu2274 | NP_532947.1 | Genebank | NCBI |
|  |  |  | *Chromobacterium violaceum* | CV_1005 | AAQ58679.1 | Genebank |  |
|  |  |  | *Chromobacterium violaceum* | CV_3677 | AAQ61339.1 | Genebank |  |
|  |  |  | *Desulfovibrio desulfuricans G20* | Dde_1511 | YP_388005.1 | Genebank |  |
|  |  |  | *Escherichia coli* | EcFieF | P69380 | Swiss Prot |  |
|  |  |  | *Escherichia coli* | EcZitB | P75757 | Swiss Prot |  |
|  |  |  | *Geobacter sulfurreducens PCA* | GSU0487 | AAR33819.1 | Genebank |  |
|  |  |  | *Geobacter sulfurreducens PCA* | GSU2613 | AAR35985.1 | Genebank |  |
|  |  |  | *Klebsiella pneumoniae* | KpFieF | Q8RR17 | Swiss Prot |  |
|  |  |  | *Magnetospirillum gryphiswaldense* | MgMamB | CAJ30127.1 | Genebank |  |
|  |  |  | *Nitrosospira multiformis ATCC 25196* | Nmul_A1744 | ABB75041.1 | Genebank |  |
|  |  |  | *Pectobacterium atrosepticum* | PaFIEF | Q6CZ45 | Swiss Prot |  |
|  |  |  | *Pectobacterium atrosepticum* | PaZITB | Q6D7E5 | Swiss Prot |  |
|  |  |  | *Pseudomonas aeruginosa* | PsaPA1297 | Q9I447 | Swiss Prot |  |
|  |  |  | *Pseudomonas fluorescens Pf-5* | PfPFL_0604 | AAY96011.1 | Genebank |  |
|  |  |  | *Ralstonia solanacearum* | RSc2772 | CAD16479.1 | Genebank |  |
|  |  |  | *Ralstonia solanacearum* | RSc3077 | CAD16786.1 | Genebank |  |
|  |  |  | *Rhodopseudomonas palustris CGA009* | RPA0220 | CAE25664.1 | Genebank |  |
|  |  |  | *Rhodopseudomonas palustris CGA009* | RPA1939 | CAE27380.1 | Genebank |  |
|  |  |  | *Rickettsia felis URRWXCal2* | RF_1320 | YP_247336.1 | Genebank |  |
|  |  |  | *Rickettsia prowazekii* | RpP34 | Q9ZCC5 | Swiss Prot |  |
|  |  |  | *Rickettsia rickettsii* | RrP34 | P21559 | Swiss Prot |  |
|  |  |  | *Salmonella typhi* | StiFieF | Q8Z2W4 | Swiss Prot |  |
|  |  |  | *Salmonella typhimurium* | StyFieF | Q8ZKR4 | Swiss Prot |  |
|  |  |  | *Salmonella typhimurium* | StyZitB | Q8ZQT3 | Swiss Prot |  |
|  |  |  | *Thiomicrospira crunogena XCL-2* | Tcr_0241 | YP_390511.1 | Genebank | DOE Joint Genome Institute |
|  |  |  | *Thiomicrospira crunogena XCL-2* | Tcr_1014 | ABB41609.1 | Genebank | DOE Joint Genome Institute |
|  |  |  | *Thiomicrospira crunogena XCL-2* | Tcr_1429 | ABB42022.1 | Genebank | DOE Joint Genome Institute |
|  |  |  | *Thiomicrospira crunogena XCL-2* | Tcr_2193 | ABB42781.1 | Genebank | DOE Joint Genome Institute |
|  |  |  | *Thiomicrospira crunogena XCL-2* | Tcr_1855 | ABB42447.1 | Genebank | DOE Joint Genome Institute |
|  |  |  | *Cupriavidus* *metallidurans CH34* | DmeF | ZP_00594243 | Genebank | DOE Joint Genome Institute |
|  |  |  | *Cupriavidus* *metallidurans CH34* | WmFieF | ZP_00593836 | Genebank | DOE Joint Genome Institute |
|  |  |  | *Cupriavidus* *metallidurans CH34* | RmCzcD | P13512 | Swiss Prot | DOE Joint Genome Institute |
|  |  |  | *Xanthomonas campestris pv. vesicatoria* | XCV1414 | YP_363145.1 | Genebank |  |
|  |  |  | *Yersinia pestis* | YpeZitB | Q8ZGY6 | Swiss Prot |  |
|  |  |  | *Yersinia pseudotuberculosis* | YpsFieF | Q66GA9 | Swiss Prot |  |
|  |  |  | *Yersinia pseudotuberculosis* | YpsZitB | Q66D85 | Swiss Prot |  |
| **Eukaryotes** |  |  |  |  |  |  |  |
|  |  | Apicomplexa | *Cryptosporidium parvum* | Cgd1_3050 | EAK88341.1 | Genebank |  |
|  |  | Entamoebidae | *Entamoeba histolytica HM-1:IMSS* | Eh152.t00010 | XP_651682.1 | Genebank | Sanger Institute |
|  |  |  | *Entamoeba histolytica HM-1:IMSS* | Eh166.t00010 | XP_651432.1 | Genebank | Sanger Institute |
|  |  |  | *Entamoeba histolytica HM-1:IMSS* | Eh393.t00004 | EAL43397.1 | Genebank | Sanger Institute |
|  |  |  | *Entamoeba histolytica HM-1:IMSS* | Eh5.t00034 | EAL51642.1 | Genebank | Sanger Institute |
|  |  |  | *Entamoeba histolytica HM-1:IMSS* | Eh99.t00008 | EAL47549.1 | Genebank | Sanger Institute |
|  | **Fungi** | |  |  |  |  |  |
|  |  | Ascomycota | *Ashbya gossypii* | AgABR129Cp | AAS50900 | Genebank | AGD |
|  |  |  | *Ashbya gossypii* | AgAFL128Cp | AAS53246 | Genebank | AGD |
|  |  |  | *Ashbya gossypii* | AgAGL102Wp | AAS54389 | Genebank | AGD |
|  |  |  | *Ashbya gossypii* | AgAGL227Wp | AAS54264 | Genebank | AGD |
|  |  |  | *Aspergillus fumigatus* | Af1g12090 | CAE47937 | Genebank | TIGR |
|  |  |  | *Aspergillus fumigatus* | Af1g14440 | BAE64878 | Genebank | TIGR |
|  |  |  | *Aspergillus fumigatus* | Af2g14570 | XP_755789 | Genebank | TIGR |
|  |  |  | *Aspergillus fumigatus* | Af4g04150 | XP_746601 | Genebank | TIGR |
|  |  |  | *Aspergillus fumigatus* | Af5g09830 | XP_753657 | Genebank | TIGR |
|  |  |  | *Aspergillus fumigatus* | Af6g00440 | XP_731511 | Genebank | TIGR |
|  |  |  | *Aspergillus fumigatus* | Af6g14170 | XP_751291 | Genebank | TIGR |
|  |  |  | *Aspergillus fumigatus* | Af7g06570 | XP_748854 | Genebank | TIGR |
|  |  |  | *Aspergillus nidulans* | AN1076.2 | EAA66194.1 | Genebank |  |
|  |  |  | *Aspergillus nidulans* | AN1146.2 | AN1146.2 | Broad Institute, v. 2 |  |
|  |  |  | *Aspergillus nidulans* | AN1795.2 | AN1795.2 | Broad Institute, v. 2 |  |
|  |  |  | *Aspergillus nidulans* | AN5049.2 | AN5049.2 | Broad Institute, v. 2 |  |
|  |  |  | *Aspergillus nidulans* | AN5347.2 | AN5347.2 | Broad Institute, v. 2 |  |
|  |  |  | *Aspergillus nidulans* | AN7036.2 | AN7036.2 | Broad Institute, v. 2 |  |
|  |  |  | *Aspergillus nidulans* | AN8791.2 | AN8791.2 | Broad Institute, v. 2 |  |
|  |  |  | *Botrytis cinerea* | BC1G_00092.1 | BC1G_00092.1 | Broad Institute, v. 1 |  |
|  |  |  | *Botrytis cinerea* | BC1G_04719.1 | BC1G_04719.1 | Broad Institute, v. 1 |  |
|  |  |  | *Botrytis cinerea* | BC1G_05581.1 | BC1G_05581.1 | Broad Institute, v. 1 |  |
|  |  |  | *Botrytis cinerea* | BC1G_13811.1 | BC1G_13811.1 | Broad Institute, v. 1 |  |
|  |  |  | *Botrytis cinerea* | BC1G_14239.1 | BC1G_14239.1 | Broad Institute, v. 1 |  |
|  |  |  | *Botrytis cinerea* | BC1G_15674.1 | BC1G_15674.1 | Broad Institute, v. 1 |  |
|  |  |  | *Candida albicans* | CaO19.376 | EAK98200 | Genebank |  |
|  |  |  | *Candida glabrata* | CAGL0G07997g | CAG59637 | Genebank |  |
|  |  |  | *Candida lipolytica* | YALI0B02552g | CAG82647 | Genebank |  |
|  |  |  | *Debaryomyces hansenii* | DEHA0C09933g | CAG86140 | Genebank |  |
|  |  |  | *Fusarium graminearum* | FG07454 | EAA77471.1 | Genebank |  |
|  |  |  | *Fusarium gramineaum* | FG00947.1 | FG00947.1 | Broad Institute, v. 1 |  |
|  |  |  | *Fusarium gramineaum* | FG01162.1 | FG01162.1 | Broad Institute, v. 1 |  |
|  |  |  | *Fusarium gramineaum* | FG01883.1 | FG01883.1 | Broad Institute, v. 1 |  |
|  |  |  | *Fusarium gramineaum* | FG05506.1 | FG05506.1 | Broad Institute, v. 1 |  |
|  |  |  | *Fusarium gramineaum* | FG05723.1 | FG05723.1 | Broad Institute, v. 1 |  |
|  |  |  | *Fusarium gramineaum* | FG09632.1 | FG09632.1 | Broad Institute, v. 1 |  |
|  |  |  | *Fusarium gramineaum* | FG09759.1 | FG09759.1 | Broad Institute, v. 4 |  |
|  |  |  | *Kluyveromyces lactis* | Kl_Q6CKZ6 | CAG98101 | Genebank |  |
|  |  |  | *Magnaporthe grisea* | MG03634.4 | MG03634.4 | Broad Institute, v. 4 |  |
|  |  |  | *Magnaporthe grisea* | MG04407.4 | MG04407.4 | Broad Institute, v. 4 |  |
|  |  |  | *Magnaporthe grisea* | MG04623.4 | MG04623.4 | Broad Institute, v. 4 |  |
|  |  |  | *Magnaporthe grisea* | MG04968.4 | MG04968.4 | Broad Institute, v. 4 |  |
|  |  |  | *Magnaporthe grisea* | MG06247.4 | MG06247.4 | Broad Institute, v. 4 |  |
|  |  |  | *Magnaporthe grisea* | MG07494.4 | MG07494.4 | Broad Institute, v. 4 |  |
|  |  |  | *Magnaporthe grisea* | MG10493.4 | MG10493.4 | Broad Institute, v. 4 |  |
|  |  |  | *Neurospora crassa* | NCU01254 | EAA31338 | Genebank |  |
|  |  |  | *Neurospora crassa* | NCU03145.2 | NCU03145.2 | Broad Institute, v. 2 |  |
|  |  |  | *Neurospora crassa* | NCU04818.2 | NCU04818.2 | Broad Institute, v. 2 |  |
|  |  |  | *Neurospora crassa* | NCU05157.2 | NCU05157.2 | Broad Institute, v. 2 |  |
|  |  |  | *Neurospora crassa* | NCU06699.2 | NCU06699.2 | Broad Institute, v. 2 |  |
|  |  |  | *Neurospora crassa* | NCU07262.2 | NCU07262.2 | Broad Institute, v. 2 |  |
|  |  |  | *Neurospora crassa* | NCU07879.2 | NCU07879.2 | Broad Institute, v. 2 |  |
|  |  |  | *Neurospora crassa* | NCU09368.2 | NCU09368.2 | Broad Institute, v. 2 |  |
|  |  |  | *Saccharomyces cerevisiae* | ScCOT1 | CAA99636 | Genebank | SGD |
|  |  |  | *Saccharomyces cerevisiae* | ScMMT1 | NP_013902 | Genebank | SGD |
|  |  |  | *Saccharomyces cerevisiae* | ScMMT2 | NP_015100 | Genebank | SGD |
|  |  |  | *Saccharomyces cerevisiae* | ScMSC2 | Q03455 | Genebank | SGD |
|  |  |  | *Saccharomyces cerevisiae* | ScZRC1 | CAA88653 | Genebank | SGD |
|  |  |  | *Saccharomyces cerevisiae* | ScZRG17 | NP_014437 | Genebank | SGD |
|  |  |  | *Schizosaccharomyces pombe* | SPAC17D4.03c | Q9HGQ3 | Swiss Prot |  |
|  |  |  | *Schizosaccharomyces pombe* | SpO14329 | O14329 | Genebank |  |
|  |  |  | *Schizosaccharomyces pombe* | SpZHF1 | O13918 | Swiss Prot |  |
|  |  |  | *Sclerotinia sclerotiorum* | SS1G_01056.1 | SS1G_01056.1 | Broad Institute, v. 1 |  |
|  |  |  | *Sclerotinia sclerotiorum* | SS1G_02998.1 | SS1G_02998.1 | Broad Institute, v. 1 |  |
|  |  |  | *Sclerotinia sclerotiorum* | SS1G_04677.1 | SS1G_04677.1 | Broad Institute, v. 1 |  |
|  |  |  | *Sclerotinia sclerotiorum* | SS1G_06298.1 | SS1G_06298.1 | Broad Institute, v. 1 |  |
|  |  |  | *Sclerotinia sclerotiorum* | SS1G_07340.1 | SS1G_07340.1 | Broad Institute, v. 1 |  |
|  |  |  | *Stagonospora nodorum* | SNU00278.1 | SNU00278.1 | Broad Institute, v. 1 |  |
|  |  |  | *Stagonospora nodorum* | SNU01554.1 | SNU01554.1 | Broad Institute, v. 1 |  |
|  |  |  | *Stagonospora nodorum* | SNU02386.1 | SNU02386.1 | Broad Institute, v. 1 |  |
|  |  |  | *Stagonospora nodorum* | SNU02584.1 | SNU02584.1 | Broad Institute, v. 1 |  |
|  |  |  | *Stagonospora nodorum* | SNU04675.1 | SNU04675.1 | Broad Institute, v. 1 |  |
|  |  |  | *Stagonospora nodorum* | SNU09283.1 | SNU09283.1 | Broad Institute, v. 1 |  |
|  |  |  | *Stagonospora nodorum* | SNU09445.1 | SNU09445.1 | Broad Institute, v. 1 |  |
|  |  |  | *Stagonospora nodorum* | SNU09657.1 | SNU09657.1 | Broad Institute, v. 1 |  |
|  |  | Basidiomycota | *Paxillus involutus* | PiMnT1 | DQ515983 | Genebank |  |
|  |  |  | *Phanerochaete chrysosporium* | Pce_gwh2.12.153.1 | e_gwh2.12.153.1 [Phchr1:124205] | DOE Joint Genome Institute, v. 2.0 | |
|  |  |  | *Phanerochaete chrysosporium* | Pce_gww2.11.228.1 | e_gww2.11.228.1 [Phchr1:134940] | DOE Joint Genome Institute, v. 2.0 | |
|  |  |  | *Phanerochaete chrysosporium* | Pce_gww2.13.213.1 | e_gww2.13.213.1 [Phchr1:135578] | DOE Joint Genome Institute, v. 2.0 | |
|  |  |  | *Phanerochaete chrysosporium* | Pce_gww2.15.88.1 | e_gww2.15.88.1 [Phchr1:140863] | DOE Joint Genome Institute, v. 2.0 | |
|  |  |  | *Ustilago maydis* | UM00151.1 | UM00151.1 | Broad Institute, v. 1 |  |
|  |  |  | *Ustilago maydis* | UM02906.1 | UM02906.1 | Broad Institute, v. 1 |  |
|  |  |  | *Ustilago maydis* | UM03814.1 | UM03814.1 | Broad Institute, v. 1 |  |
|  |  |  | *Ustilago maydis* | UM05647.1 | UM05647.1 | Broad Institute, v. 1 |  |
|  |  |  | *Ustilago maydis* | UM06139.1 | UM06139.1 | Broad Institute, v. 1 |  |
|  |  | Glomeromycota | *Glomus intraradices* | GintZnT1 | CAE00445.1 | Genebank |  |
|  |  | Zygomycota | *Rhizopus oryzae* | RO3G_02565.1 | RO3G_02565.1 | Broad Institute, v. 1 |  |
|  |  |  | *Rhizopus oryzae* | RO3G_02629.1 | RO3G_02629.1 | Broad Institute, v. 1 |  |
|  |  |  | *Rhizopus oryzae* | RO3G_08509.1 | RO3G_08509.1 | Broad Institute, v. 1 |  |
|  |  |  | *Rhizopus oryzae* | RO3G_09126.1 | RO3G_09126.1 | Broad Institute, v. 1 |  |
|  |  |  | *Rhizopus oryzae* | RO3G_09636.1 | RO3G_09636.1 | Broad Institute, v. 1 |  |
|  | **Metazoa** | |  |  |  |  |  |
|  |  | Arthropoda | *Drosophila melanogaster* | DmCG11163-PA | AAF57308.2 | Genebank | FlyBase |
|  |  |  | *Drosophila melanogaster* | DmCG17723-PA | AAF47755.1 | Genebank | FlyBase |
|  |  |  | *Drosophila melanogaster* | DmCG3994-PA | AAF53443.3 | Genebank | FlyBase |
|  |  |  | *Drosophila melanogaster* | DmCG3994-PB | AAN10893.1 | Genebank | FlyBase |
|  |  |  | *Drosophila melanogaster* | DmCG6672-PA | AAF54604.1 | Genebank | FlyBase |
|  |  | Chordata | *Gallus gallus* | GgZnT6 | AAY53770.1 | Genebank |  |
|  |  |  | *Homo sapiens* | HsZnT1 | Q9Y6M5 | Swiss Prot | NCBI |
|  |  |  | *Homo sapiens* | HsZnT10a | NP_061183.2 | Genebank | NCBI |
|  |  |  | *Homo sapiens* | HsZnT10b | NP_001004433.1 | Genebank | NCBI |
|  |  |  | *Homo sapiens* | HsZnT2 | NP_001004434.1 | Genebank | NCBI |
|  |  |  | *Homo sapiens* | HsZnT3 | Q99726 | Swiss Prot | NCBI |
|  |  |  | *Homo sapiens* | HsZnT4 | O14863 | Swiss Prot | NCBI |
|  |  |  | *Homo sapiens* | HsZnT5 | AAM09099.1 | Genebank | NCBI |
|  |  |  | *Homo sapiens* | HsZnT6 | NP_060434.2 | Genebank | NCBI |
|  |  |  | *Homo sapiens* | HsZnT7 | AAM21969.1 | Genebank | NCBI |
|  |  |  | *Homo sapiens* | HsZnT8 | NP_776250.2 | Genebank | NCBI |
|  |  |  | *Homo sapiens* | HsZnT9 | NP_006336.3 | Genebank | NCBI |
|  |  |  | *Homo sapiens* | HsZTL1 | AAL84188.1 | Genebank | NCBI |
|  |  |  | *Macaca fascicularis* | MacfZnT1 | Q4R6K2 | Swiss Prot |  |
|  |  |  | *Mus musculus* | MmZnT1 | Q60738 | Swiss Prot |  |
|  |  |  | *Mus musculus* | MmZnT3 | P97441 | Swiss Prot |  |
|  |  |  | *Mus musculus* | MmZnT3-1 | NP_035903.1 | Genebank |  |
|  |  |  | *Mus musculus* | MmZnT4 | O35149 | Swiss Prot |  |
|  |  |  | *Mus musculus* | MmZnT6 | NP_659047.2 | Genebank |  |
|  |  |  | *Rattus norvegicus* | RnZnT1 | Q62720 | Swiss Prot |  |
|  |  |  | *Rattus norvegicus* | RnZnT2 | Q62941 | Swiss Prot |  |
|  |  |  | *Rattus norvegicus* | RnZnT4 | O55174 | Swiss Prot |  |
|  |  |  | *Takifugu rubripes* | TrZnT-1 | NP_001027895.1 | Genebank |  |
|  |  | Nematoda | *Caenorhabditis elegans* | Ce F56C9.3 | AAK18959.2 | Genebank | NCBI |
|  |  |  | *Caenorhabditis elegans* | CeCDF1a | AAK39165.1 | Genebank | NCBI |
|  |  |  | *Caenorhabditis elegans* | CeCDF1b | AAL13323.1 | Genebank | NCBI |
|  |  |  | *Caenorhabditis elegans* | CeF41C6.7 | AAA80448.2 | Genebank | NCBI |
|  |  |  | *Caenorhabditis elegans* | CePDB1.1 | AAA81718.3 | Genebank | NCBI |
|  | **Viridiplanta** | |  |  |  |  | |
|  |  | Chlorophyta | *Chlamydomonas reinhardtii* | CrMTP1 | 670027 | DOE Joint Genome Institute, v. 2.0 | |
|  |  |  | *Chlamydomonas reinhardtii* | CrMTP2 | 110062 | DOE Joint Genome Institute, v. 2.0 | |
|  |  |  | *Chlamydomonas reinhardtii* | CrMTP3 | 110067 | DOE Joint Genome Institute, v. 2.0 | |
|  |  |  | *Chlamydomonas reinhardtii* | CrMTP4 | 110212 | DOE Joint Genome Institute, v. 2.0 | |
|  |  |  | *Chlamydomonas reinhardtii* | CrMTP5 | 290110 | DOE Joint Genome Institute, v. 2.0 | |
|  |  | Streptophyta | *Arabidopsis halleri* | AhCDF1-3 | CAD89013.1 | Genebank |  |
|  |  |  | *Arabidopsis lyrata* | AlMTP1-1 | CAG28982.1 | Genebank |  |
|  |  |  | *Arabidopsis thaliana* | AtMTP1 | NP_182203.1 | Genebank | TIGR |
|  |  |  | *Arabidopsis thaliana* | AtMTP10 | NP_173081.2 | Genebank | TIGR |
|  |  |  | *Arabidopsis thaliana* | AtMTP11 | NP_181477.1 | Genebank | TIGR |
|  |  |  | *Arabidopsis thaliana* | AtMTP12 | NP_178539.2 | Genebank | TIGR |
|  |  |  | *Arabidopsis thaliana* | AtMTP2 | NP_191753.1 | Genebank | TIGR |
|  |  |  | *Arabidopsis thaliana* | AtMTP3 | NP_191440.2 | Genebank | TIGR |
|  |  |  | *Arabidopsis thaliana* | AtMTP4 | NP_180502.2 | Genebank | TIGR |
|  |  |  | *Arabidopsis thaliana* | AtMTP5 | NP_187817.2 | Genebank | TIGR |
|  |  |  | *Arabidopsis thaliana* | AtMTP6 | NP_182304.2 | Genebank | TIGR |
|  |  |  | *Arabidopsis thaliana* | AtMTP7 | NP_564594.1 | Genebank | TIGR |
|  |  |  | *Arabidopsis thaliana* | AtMTP8 | NP_191365.2 | Genebank | TIGR |
|  |  |  | *Arabidopsis thaliana* | AtMTP9 | NP_178070.2 | Genebank | TIGR |
|  |  |  | *Nicotiana glauca* | NgMTP1 | BAD89561.1 | Genebank |  |
|  |  |  | *Nicotiana tabacum* | NtMTP1A | BAD89562.1 | Genebank |  |
|  |  |  | *Nicotiana tabacum* | NtMTP1B | BAD89563.1 | Genebank |  |
|  |  |  | *Oryza sativa* | Os01g03910 | BAA99362 | Genebank |  |
|  |  |  | *Oryza sativa* | Os01g03914 | Os01g03914 | TIGR | TIGR |
|  |  |  | *Oryza sativa* | Os01g62070 | Os01g62070 | TIGR | TIGR |
|  |  |  | *Oryza sativa* | Os02g53490 | Os02g53490 | TIGR | TIGR |
|  |  |  | *Oryza sativa* | Os03g12530 | Os03g12530 | TIGR | TIGR |
|  |  |  | *Oryza sativa* | Os03g22550 | Os03g22550 | TIGR | TIGR |
|  |  |  | *Oryza sativa* | Os05g03780 | Os05g03780 | TIGR | TIGR |
|  |  |  | *Oryza sativa* | Os05g38670 | Os05g38670 | TIGR | TIGR |
|  |  |  | *Oryza sativa* | Os08g32650 | Os08g32650 | TIGR | TIGR |
|  |  |  | *Oryza sativa* | OsJNBa0035B13 | CAD40428.3 | Genebank |  |
|  |  |  | *Populus trichocarpa* | PtrMTP1 | ESTEXT_FGENESH4_PM.C_LG_X0839 | DOE Joint Genome Institute, v. 1.1 | |
|  |  |  | *Populus trichocarpa* | PtrMTP10 | GW1.I.6617.1 | DOE Joint Genome Institute, v. 1.1 | |
|  |  |  | *Populus trichocarpa* | PtrMTP11.1 | GW1.I.5578.1 | DOE Joint Genome Institute, v. 1.1 | |
|  |  |  | *Populus trichocarpa* | PtrMTP11.2 | FGENESH4_PG.C_LG_VIII000724 | DOE Joint Genome Institute, v. 1.1 | |
|  |  |  | *Populus trichocarpa* | PtrMTP12 | EUGENE3.00111272 | DOE Joint Genome Institute, v. 1.1 | |
|  |  |  | *Populus trichocarpa* | PtrMTP2 | FGENESH4_PM.C_LG_II000836 | DOE Joint Genome Institute, v. 1.1 | |
|  |  |  | *Populus trichocarpa* | PtrMTP3.1 | GW1.XIV.294.1 | DOE Joint Genome Institute, v. 1.1 | |
|  |  |  | *Populus trichocarpa* | PtrMTP3.2 | GW1.I.6615.1 | DOE Joint Genome Institute, v. 1.1 | |
|  |  |  | *Populus trichocarpa* | PtrMTP4.1 | GW1.18419.4.1 | DOE Joint Genome Institute, v. 1.1 | |
|  |  |  | *Populus trichocarpa* | PtrMTP4.2 | FGENESH4_PM.C_SCAFFOLD_57000020 | DOE Joint Genome Institute, v. 1.1 | |
|  |  |  | *Populus trichocarpa* | PtrMTP6 | FGENESH4_PM.C_LG_X000667 | DOE Joint Genome Institute, v. 1.1 | |
|  |  |  | *Populus trichocarpa* | PtrMTP7 | GW1.X.3157.1 | DOE Joint Genome Institute, v. 1.1 | |
|  |  |  | *Populus trichocarpa* | PtrMTP8.1 | EUGENE3.00080435 | DOE Joint Genome Institute, v. 1.1 | |
|  |  |  | *Populus trichocarpa* | PtrMTP8.2 | GW1.I.1107.1 | DOE Joint Genome Institute, v. 1.1 | |
|  |  |  | *Populus trichocarpa* | PtrMTP8.4 | FGENESH4_PM.C_LG_III000766 | DOE Joint Genome Institute, v. 1.1 | |
|  |  |  | *Populus trichocarpa* | PtrMTP9 | ESTEXT_GENEWISE1_V1.C_LG_XIV1924 | DOE Joint Genome Institute, v. 1.1 | |
|  |  |  | *Populus trichocarpa x Populus deltoides* | PtdMTP1 | AAR23528.1 | Genebank |  |
|  |  |  | *Stylosanthes hamata* | ShMTP1 | AY181256 | Genebank |  |
|  |  |  | *Stylosanthes hamata* | ShMTP2 | AY181257 | Genebank |  |
|  |  |  | *Stylosanthes hamata* | ShMTP3 | AY181258 | Genebank |  |
|  |  |  | *Stylosanthes hamata* | ShMTP4 | AY181259 | Genebank |  |
|  |  |  | *Thlaspi goesingense* | TgMTP1a | AAS67024.1 | Genebank |  |
|  |  |  | *Thlaspi goesingense* | TgMTP1b | AAS67025.1 | Genebank |  |
|  |  |  | *Thlaspi goesingense* | TgMTP1c | AAS67026.1 | Genebank |  |
